# Insight Into Distinct Functional Roles of the Flagellar ATPase Complex for Flagellar Assembly in *Salmonella*

**DOI:** 10.3389/fmicb.2022.864178

**Published:** 2022-05-04

**Authors:** Tohru Minamino, Miki Kinoshita, Keiichi Namba

**Affiliations:** ^1^Graduate School of Frontier Biosciences, Osaka University, Osaka, Japan; ^2^RIKEN SPring-8 Center and Center for Biosystems Dynamics Research, Osaka, Japan; ^3^JEOL YOKOGUSHI Research Alliance Laboratories, Osaka University, Osaka, Japan

**Keywords:** ATPase, bacterial flagella, F_0_F_1_ ATP synthase, flagellar assembly, proton motive force (pmf), protein translocation, type III secretion system (T3SS)

## Abstract

Most motile bacteria utilize the flagellar type III secretion system (fT3SS) to construct the flagellum, which is a supramolecular motility machine consisting of basal body rings and an axial structure. Each axial protein is translocated via the fT3SS across the cytoplasmic membrane, diffuses down the central channel of the growing flagellar structure and assembles at the distal end. The fT3SS consists of a transmembrane export complex and a cytoplasmic ATPase ring complex with a stoichiometry of 12 FliH, 6 FliI and 1 FliJ. This complex is structurally similar to the cytoplasmic part of the F_O_F_1_ ATP synthase. The export complex requires the FliH_12_-FliI_6_-FliJ_1_ ring complex to serve as an active protein transporter. The FliI_6_ ring has six catalytic sites and hydrolyzes ATP at an interface between FliI subunits. FliJ binds to the center of the FliI_6_ ring and acts as the central stalk to activate the export complex. The FliH dimer binds to the N-terminal domain of each of the six FliI subunits and anchors the FliI_6_-FliJ_1_ ring to the base of the flagellum. In addition, FliI exists as a hetero-trimer with the FliH dimer in the cytoplasm. The rapid association-dissociation cycle of this hetero-trimer with the docking platform of the export complex promotes sequential transfer of export substrates from the cytoplasm to the export gate for high-speed protein transport. In this article, we review our current understanding of multiple roles played by the flagellar cytoplasmic ATPase complex during efficient flagellar assembly.

## Introduction

**FIGURE 1:**
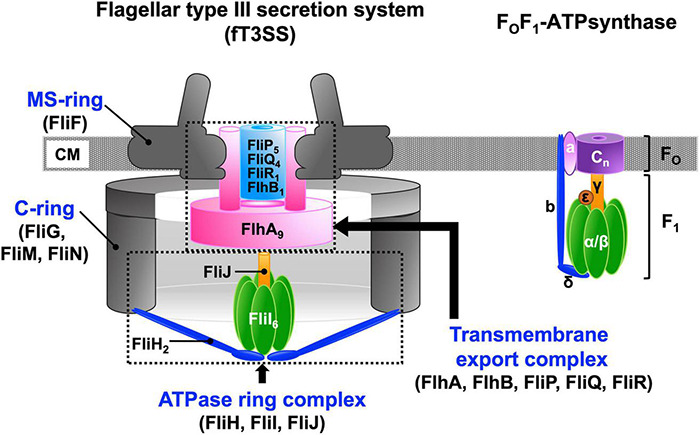
Schematic diagrams of the flagellar type III export apparatus and F_O_F ATP synthase. The flagellar type III secretion system (fT3SS) is composed of five membrane proteins, FlhA, FlhB, FliP, FliQ, and FliR and three cytoplasmic proteins, FliH, FliI, and FliJ. FlhA, FlhB, FliP, FliQ and FliR assembles into a transmembrane export complex within the MS-ring of the basal body of the flagellum. FliH, FliI, and FliJ form a cytoplasmic ATPase ring. The FliI_6_-FliJ_1_ ring complex is structurally similar to the α_3_β_3_γ_1_ ring complex of the F_O_F_1_ ATP synthase. The N-terminal and C-terminal domains of FliH structurally are similar in structure to the b and δ subunits, respectively, of the F_O_F_1_ ATP synthase. The FliH dimer acts as a peripheral stalk that anchors the FliI_6_-FliJ ring complex to the base of the flagellum in a similar manner as the b and δ subunits of the F_O_F1 ATP synthase connect the α_3_β_3_γ ring complex to membrane-embedded F_O_. The stoichiometry of the c-ring varies dramatically from c_8_ up to at least c_15_. CM, cytoplasmic membrane.

Pathogenic bacteria use virulence-associated type III secretion systems (vT3SS), also known as the injectisomes, to inject virulence effector proteins directly into eukaryotic host cells as part of their infection process. Motile bacteria employ the flagellar type III secretion system (fT3SS) to construct a supramolecular motility machine, the flagellum, on the cell surface (Wagner and Diepold, 2020). A remarkable feature of both the vT3SS and fT3SS is that the protein export apparatus is capable of translocating export substrates across the cytoplasmic membrane at a rate of tens of thousands of amino acids per second (Iino, 1974; Chen et al., 2017; Renault et al., 2017). The protein export apparatus of the T3SS is composed of a transmembrane export complex powered by the proton motive force (PMF) across the cytoplasmic membrane and a cytoplasmic ATPase ring complex (Figure 1). The transmembrane export complex is composed of five conserved membrane proteins: FlhA, FlhB, FliP, FliQ, and FliR in the fT3SS; SctV, SctU, SctR, SctS, and SctT in the vT3SS. The cytoplasmic ATPase ring complex is composed of three cytoplasmic proteins, FliH, FliI, and FliJ in the fT3SS and SctL, SctN, and SctO in the vT3SS. The ATPase ring complex is structurally similar to the cytoplasmic part of the F_O_F_1_ ATP synthase, which is a rotary motor that couples proton (H^+^) flow through F_O_ with ATP synthesis by F_1_ (Minamino, 2014; Minamino et al., 2020b).

**FIGURE 2:**
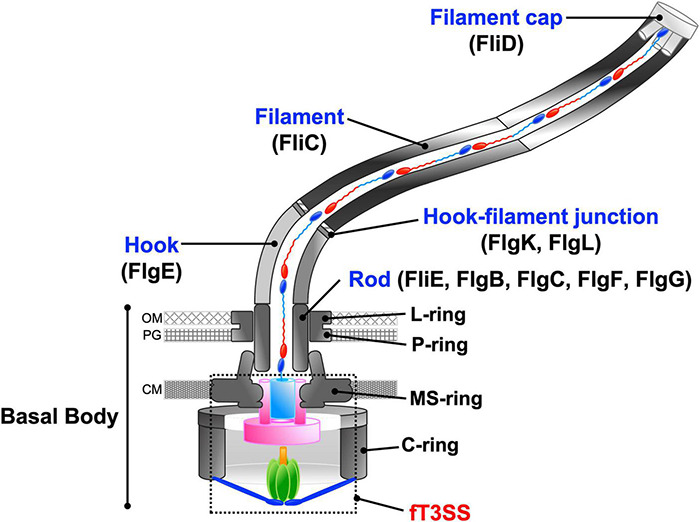
Schematic diagram of the bacterial flagellum. The bacterial flagellum is composed of basal body rings, namely the C-ring, MS-ring, L-ring, and P-ring, and an axial structure consisting of the rod, the hook, the hook-filament junction, the filament, and the filament cap. To construct the axial structure beyond the cytoplasmic membrane, flagellar axial proteins are translocated through the fT3SS, diffuse down a narrow central channel, and assemble at the tip of the growing structure. OM, outer membrane; PG, peptidoglycan layer; CM, cytoplasmic membrane.

The flagellum of *Salmonella enterica* serovar Typhimurium (hereafter referred to as *Salmonella*) is composed of about 30 different proteins whose copy numbers range from a few to tens of thousands. The *Salmonella* flagellum is divided into three main structural parts: the basal body, the hook, and the filament (Figure 2). The basal body is located within the cell envelop and serves as a bi-directional rotary motor fueled by the PMF across the cytoplasmic membrane. The hook and filament extend into the cell exterior. The filament functions as a helical propeller to produce the thrust that pushes the cell body forward. The hook between the basal body and filament acts as a universal joint to transmit torque produced by the motor to the filament (Nakamura and Minamino, 2019).

The axial structure of the *Salmonella* flagellum is composed of the rod (FliE, FlgB, FlgC, FlgF, FlgG), the hook (FlgE), the hook-filament junction (FlgK, FlgL), the filament (flagellin, FliC or FljB) and the filament cap (FliD) (Figure 2). The assembly of the axial structure begins with the rod, followed by the hook with the help of the hook cap (FlgD). Upon completion of hook assembly, the hook cap is replaced by FlgK, and then FlgK and FlgL self-assemble into the hook-filament junction structure at the hook tip. FliD forms the filament cap at the tip of the junction structure and promotes the assembly of newly transported flagellin molecules into the long helical filament (Macnab, 2003).

To construct the axial structure beyond the cellular membranes, fourteen different proteins are translocated across the cytoplasmic membrane via the fT3SS, diffuse down the narrow central channel, and assemble at the tip of the growing structure (Figure 2). They can be classified into two export classes: one is the rod-type (FliE, FlgB, FlgC, FlgF, FlgG, FlgJ) and hook-type (FlgD, FlgE, FliK) class needed for assembly of the rod and hook. The other is the filament-type class (FlgK, FlgL, FlgM, FliC, FliD) responsible for filament assembly. The fT3SS secrets a molecular ruler protein, FliK, to measure the length of the hook during hook assembly and switches its substrate specificity from rod/hook-type proteins to filament-type proteins when the hook reaches its mature length of about 55 nm. At that point hook assembly terminates and filament assembly initiates (Minamino, 2018).

The fT3SS and vT3SS utilize the PMF across the cytoplasmic membrane and ATP hydrolysis to drive protein translocation across the cytoplasmic membrane (Minamino and Namba, 2008; Paul et al., 2008; Lee et al., 2014). The *Salmonella* fT3SS has a backup engine powered by a sodium (Na^+^) motive force (SMF) across the cytoplasmic membrane to continue flagellar assembly when the cytoplasmic ATPase ring complex does not work properly, as during biofilm development (Minamino et al., 2016b,2021a).

Once the transmembrane export complex of the *Salmonella* fT3SS is activated by ATP hydrolysis in the cytoplasmic ATPase ring complex, it becomes an active H^+^/protein antiporter that couples inward-directed H^+^ flow with outward-directed protein export (Minamino et al., 2011). Furthermore, the cytoplasmic ATPase complex allows the export complex to coordinate flagellar protein export with assembly in *Salmonella* (Minamino et al., 2016a; Inoue et al., 2018). Thus, the cytoplasmic ATPase ring complex acts as an activator of the H^+^-driven export engine and also contributes to efficient and robust protein export by the export complex. This review describes our current understanding of the structure and function of the flagellar cytoplasmic ATPase complex in *Salmonella*.

### Structure and Function of the Transmembrane Export Complex

The transmembrane export complex of the fT3SS is located inside the MS-ring formed by the transmembrane protein FliF (Figure 1; Johnson et al., 2021; Kawamoto et al., 2021; Takekawa et al., 2021; Tan et al., 2021). It consists of nine copies of FlhA, a single copy of FlhB, five copies of FliP, four copies of FliQ, and a single copy of FliR (Abrusci et al., 2013; Kuhlen et al., 2018, 2020; Johnson et al., 2019).

**FIGURE 3:**
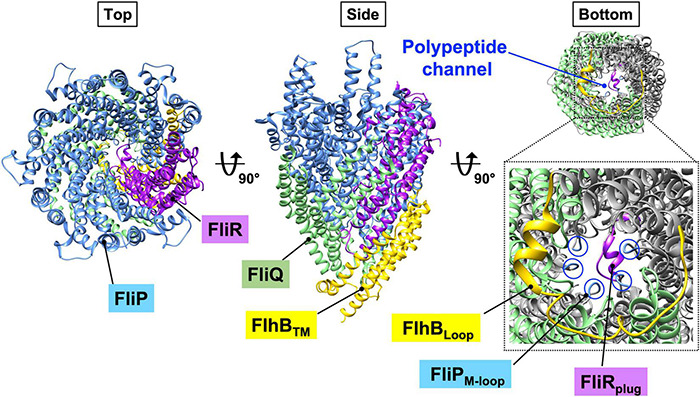
CryoEM structure of the FlhB_1_-FliP_5_-FliQ_4_-FliR_1_ complex (PDB ID: 6S3L). FliP and FliR assemble into the FliP_5_-FliR_1_ complex with the help of the flagellum-specific transmembrane protein, FliO. Four copies of FliQ associates with the outside of the FliP_5_-FliR_1_ complex. The central pore of the FliP_5_-FliR_1_ complex is thought to be a polypeptide channel. The FliP_5_-FliQ_4_-FliR_1_ complex adopts a right-handed helix similar to that of the flagellar axial structure. The transmembrane domain of FlhB (FlhB_TM_) associates with the FliP_5_-FliQ_4_-FliR_1_ complex. The highly conserved M-loop formed by Met-209, Met-210, and Met-211 of FliP (FliP_M–loop_) and the plug loop composed of residues 106–122 of FliR (FliR_plug_) block leakage of any small molecules during protein translocation. The cytoplasmic loop of FlhB FlhB_Loop_) interacts with all four FliQ subunits. Because the entrance gate of the FlhB_1_-FliP_5_-FliQ_4_-FliR_1_ complex is closed, FlhB is proposed to regulate opening of the gate to the polypeptide channel. Cyan, FliP; green, FliQ; magenta, FliR; yellow, FlhB_TM_.

FliP and FliR assemble into the FliP_5_-FliR_1_ complex with the help of the FliO scaffolding protein and form the polypeptide channel for the translocation of export substrates across the cytoplasmic membrane (Figure 3, left panel) (Fabiani et al., 2017; Fukumura et al., 2017). Four FliQ subunits bind to the outside of the FliP_5_-FliR_1_ complex to form the FliP_5_-FliQ_4_-FliR_1_ complex (Figure 3, middle panel). A flexible loop formed by the highly conserved Met-209, Met-210, and Met-211 residues of FliP (the M-loop) on the cytoplasmic side of the polypeptide channel and a plug loop composed of residues 106–122 of FliR (the R-plug) seem to prevent the leakage of small molecules during high-speed protein translocation (Figure 3, right panel) (Ward et al., 2018; Hüsing et al., 2021). The FliP_5_-FliQ_4_-FliR_1_ complex has a helical arrangement of subunits similar to the rod (Figure 3), so FliE, which is the first export substrate transported by the fT3SS (Minamino and Macnab, 1999; Minamino et al., 2000), can directly assemble at the distal end of the FliP_5_-FliQ_4_-FliR_1_ complex to form the most proximal part of the rod. Interactions between FliE and FliF not only firmly connect the rod with the MS ring but also open the exit gate of the polypeptide channel through conformational changes of FliP and FliR (Hendriksen et al., 2021).

*Salmonella* FlhB consists of an N-terminal transmembrane domain (FlhB_TM_) with four transmembrane helices (TMHs) (residues 1–211) and a large C-terminal cytoplasmic domain (FlhB_C_) (residues 212–383) (Minamino et al., 1994; Kinoshita et al., 2021). FlhB_TM_ associates with the FliP_5_-FliQ_4_-FliR_1_ complex to form the FliP_5_-FliQ_4_-FliR_1_-FlhB_1_ complex (Figure 3, middle panel), and the cytoplasmic loop connecting TMH-2 and TMH-3 (FlhB_Loop_) wraps around the entrance gate of the FliP_5_-FliQ_4_-FliR_1_ complex through interactions of the loop with each FliQ subunit (Figure 3, right panel). It is thus plausible that FlhB may coordinate gate opening for substrate entry into the polypeptide channel. Recent genetic analysis has suggested that the N-terminal cytoplasmic tail of FlhB and FlhB_C_ are involved, along with the cytoplasmic ATPase complex, in the gating function of FlhB (Kinoshita et al., 2021).

**FIGURE 4:**
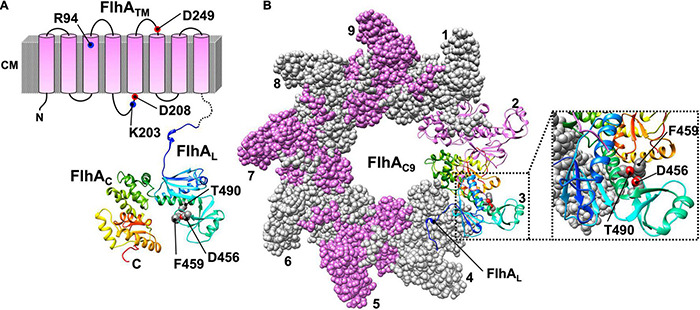
Atomic model of the cytoplasmic domain of FlhA (PDB ID: 3A5I). **(A)** Topological model of FlhA. FlhA is composed of an N-terminal transmembrane region with eight transmembrane helices (FlhA_TM_) and a large C-terminal cytoplasmic domain (FlhA_C_). FlhA_TM_ acts as a dual-ion channel that can conduct both H^+^ and Na^+^. The highly conserved charged residues R94, K203, D208 and D249 are involved in H^+^-coupled protein export. The highly conserved residues D456, F459 and T490 of FlhA_C_ are critical for substrate recognition. A flexible linker region of FlhA (FlhA_L_), which connects FlhA_C_ with FlhA_TM_, is involved in the interaction with FliJ. The interaction between FlhA_L_ and FliJ activates the FlhA ion channel. **(B)** Model of the FlhA_C_-ring. FlhA_C_ forms a homo-nonameric ring structure. The C-terminal part of FlhA_L_ binds to the neighboring FlhA_C_ subunit to stabilize the open conformation of FlhA_C_, allowing flagellar export chaperones in complex with their cognate substrates to bind to a chaperone-binding site of FlhA_C_, which includes the D456, F459, and T490 residues.

*Salmonella* FlhA is divided into two distinct regions: an N-terminal transmembrane region (FlhA_TM_) with eight TMHs (residues 1–327) and a large C-terminal cytoplasmic region (residues 328–692) (Figure 4A; Minamino et al., 1994; Kinoshita et al., 2021). The crystal structure of the C-terminal cytoplasmic region is composed of a compactly folded domain (FlhA_C_, residues 362–692) and a flexible linker (FlhA_L_, residues 328–361) connecting FlhA_C_ with FlhA_TM_ (Figure 4A; Saijo-Hamano et al., 2010). FlhA assembles into a homo-nonamer through intermolecular interactions between FlhA_C_ subunits, and the interactions of FlhA_L_ with its neighboring FlhA_C_ subunit stabilize the FlhA_C_-ring structure (Figure 4B; Terahara et al., 2018; Kuhlen et al., 2021). FlhA_TM_ associates not only with the FliP_5_-FliQ_4_-FliR_1_ complex but also with the MS-ring (Kihara et al., 2001).

If either MS-ring or the FliP_5_FliQ_4_FliR_1_ complex is missing in *Salmonella* cells, FlhA cannot efficiently form the oligomer at the flagellar base as monitored with FlhA labeled with yellow fluorescent protein (YFP), suggesting that FlhA assembles into the export complex along with other export-gate proteins during MS-ring formation (Morimoto et al., 2014). The highly conserved Arg-94, Lys-203, Asp-208, and Asp-249 residues of FlhA_TM_ are critical for H^+^-coupled protein export (Hara et al., 2011; Erhardt et al., 2017). Over-expression of FlhA in *Escherichia coli* decreases the intracellular pH. Furthermore, over-expression of FlhA increases intracellular Na^+^ concentration in the presence of 100 mM NaCl. These observations suggest that FlhA forms a pathway for the transit of both H^+^ and Na^+^ across the cytoplasmic membrane. The *flhA(D208A)* mutation facilitates the H^+^-channel activity of FlhA, suggesting that Asp-208 of FlhA may coordinate H^+^ flow though the FlhA channel with protein export. However, this mutation does not affect the Na^+^-channel activity of FlhA at all, suggesting that the Na^+^ pathway is different from the H^+^ pathway (Minamino et al., 2016b).

FlhA_C_ and FlhB_C_ project into the cytoplasmic cavity of the basal body C-ring and form a docking platform for the cytoplasmic ATPase complex, flagellar export chaperones, and export substrates (Minamino and Macnab, 2000c; Minamino et al., 2003, 2010, 2012a; Bange et al., 2010). The FlhA_C_-FlhB_C_ docking platform determines the order of substrate export to facilitate efficient flagellar assembly and also regulates gate opening of the FlhA ion channel and the FliP_5_-FliQ_4_-FliR polypeptide channel (Minamino and Macnab, 2000a; Kinoshita et al., 2013; Inoue et al., 2019; Minamino et al., 2020a,2021b).

A highly conserved hydrophobic dimple including Phe-459, Asp-456, and Thr-490 of FlhA is critical for substrate recognition by the fT3SS during flagellar assembly (Figure 4A; Xing et al., 2018). The C-terminal part of FlhA_L_ binds to its neighboring FlhA_C_ subunit to stabilize the open conformation of FlhA_C_ in the nonameric ring, allowing flagellar export chaperones associated with their cognate substrates to bind to the conserved hydrophobic dimple with a nanomolar affinity (Inoue et al., 2021).

### Catalytic Mechanism of the FliI_6_-FliJ_1_ Ring Complex

The F_1_ ATPase is composed of three copies of the α subunit, three copies of the β subunit, a single copy of the γ subunit and a single copy of the ε subunit (Figure 1). The α and β subunits form a hetero-hexameric α_3_β_3_ ring, and the γ subunit binds within the central pore of the α_3_β_3_ ring (Abrahams et al., 1994). The ε subunit binds to the γ subunit to control the ATP hydrolysis activity of the F_1_ ATPase in an ATP-dependent manner (Kato-Yamada et al., 2000). The α_3_β_3_γ_1_ subcomplex is the minimum unit that can function as an ATP-driven rotary motor to couple ATP hydrolysis with the rotation of the γ subunit within the α_3_β_3_ ring. ATP binds to three catalytic sites in the α_3_β_3_ ring, each of which is located at an interface between the α and β subunits. Three catalytic β subunits in the α_3_β_3_ ring undergo highly cooperative and sequential conformational changes in their C-terminal domains during ATP hydrolysis. These conformational changes drive the γ subunit to rotate within the α_3_β_3_ ring (Watanabe and Noji, 2013). The FliI_6_-FliJ_1_ subcomplex of the fT3SS, which looks similar to the α_3_β_3_γ_1_ subcomplex, can act as the ATPase at the base of the flagellum (Figure 1; Ibuki et al., 2011).

**FIGURE 5:**
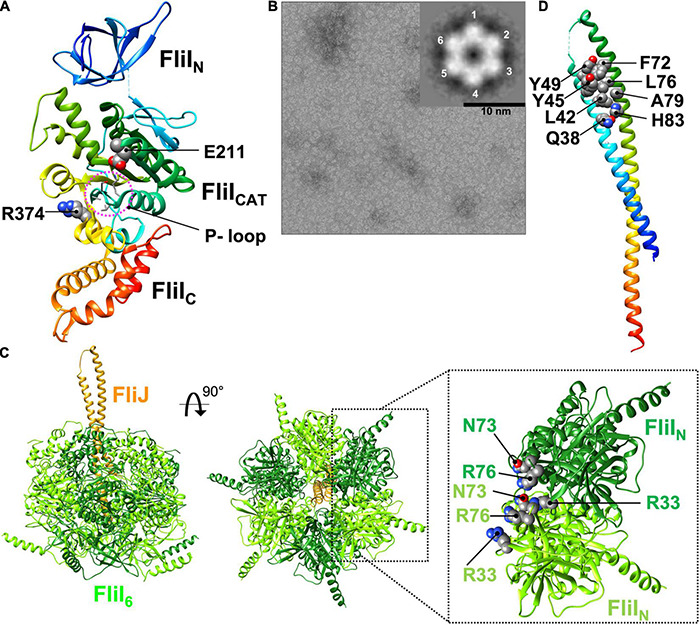
Atomic model of the FliI_6_-FliJ_1_ ATPase ring complex. **(A)** Cα ribbon representation of FliI (PDB ID: 5B0O). FliI consists of an N-terminal (FliI_N_), an ATPase (FliI_CAT_), and a C-terminal (FliI_C_) domain. FliI_N_ is involved in formation of the FliI_6_ ring. FliI_CAT_ contains the highly conserved P-loop, the catalytic glutamate (E211), and an arginine finger (R374), which are all involved in ATP hydrolysis. The ATP catalytic cycle induces sequential and cooperative conformational changes of FliI_C_, which interacts with FliJ. **(B)** Electron micrograph of negatively stained FliI ring-like structures in complex with Mg^2+^-ADP-AlF_4_. The inset shows a 2D class average of the FliI ring structure. **(C)** Model of the FliI_6_-FliJ_1_ ring model. R33, N73, and R76 of FliI_N_ regulate FliI ring formation. FliJ binds to the center of the FliI_6_ ring. **(D)** Cα ribbon representation of FliJ (PDB ID: 3AJW). FliJ forms a two stranded coiled-coil structure. The highly conserved Q38, L42, Y45, Y49, F72, L76, A79 and H83 residues of FliJ extends out of the FliI_6_ ring and are interact with FlhA_L_.

FliI is the flagellum-specific ATPase. It has highly conserved Walker A and B motifs (Vogler et al., 1991; Fan and Macnab, 1996). *Salmonella* FliI consists of three domains: N-terminal (residues 2–97, FliI_N_), ATPase (residues 109–380, FliI_CAT_) and C-terminal (residues 381–456, FliI_C_) (Figure 5A; Imada et al., 2007). Residues 98–105, most of which are invisible in the electron density map, form a flexible hinge connecting FliI_N_ and FliI_CAT_, and this flexible hinge loop undergoes conformational changes during ATP binding and hydrolysis (Minamino et al., 2001).

The structures of FliI and its fT3SS homolog SctN are remarkably similar to the α and β subunits of the F_1_ ATPase (Zarivach et al., 2007). However, in contrast to the F_1_ ATPase, FliI and SctN form homo-hexamers in an ATP-dependent manner (Figure 5B, C), and both hexamers themselves can hydrolyze ATP at the interface between FliI/SctN subunits (Claret et al., 2003; Kazetani et al., 2009). Thus, the ATPase ring complex of the T3SS has six catalytic sites. The FliI_6_ and SctN_6_ ring structures have been identified at the base of the flagellum and injectisome, respectively, by electron cryotomography and sub-tomogram averaging (Chen et al., 2011; Kawamoto et al., 2013).

Intermolecular interactions between FliI_N_ domains are required for FliI ring formation (Figure 5C; Okabe et al., 2009). The core structure of FliI_N_ can be superimposed onto the N-terminal domains of the α and β subunits of the F_1_ ATPase within α_3_β_3_ hetero-hexamer. In the FliI_6_-ring model, which was generated by fitting the crystal structure of FliI into the structures of the α and β subunits, FliI_N_ shows steric hindrance at the subunit interfaces, suggesting that a conformational change in FliI_N_ is required for FliI ring formation. Deletion of residues 2–7 of FliI_N_ suppresses FliI hexamerization and decreases the ATPase activity of FliI (Minamino et al., 2006), suggesting that the extreme N-terminal region of FliI regulates FliI oligomerization. Recently, it has been reported that Arg-33, Asn-73, and Arg-76 are also responsible for well-regulated FliI ring formation (Figure 5C; Kinoshita et al., 2021).

Amino acid residues in the F_1_ ATPase that are known to be involved in ATP hydrolysis are highly conserved in the FliI/SctN family. FliI_CAT_ contains the highly conserved P-loop (residues 182–188), the catalytic glutamate (Glu-211), and the arginine finger (Arg-374) (Figure 5A; Walker, 2013). ADP binds to the P-loop of FliI, as it does in the F_1_ ATPase. The carboxyl group of Glu-190 in the β subunit of the thermophilic *Bacillus* F_1_ ATPase, which corresponds to Glu-211 of FliI, polarizes a water molecule for the nucleophilic attack on the γ-phosphate of ATP, and the G190Q substitution results in a complete loss of ATPase activity (Shimabukuro et al., 2003). The *fliI(E211Q)* mutation completely abolishes ATPase activity but does not affect the binding of ATP to the P-loop, and FliI with the E211Q substitution can form the hexamer ring in the presence of Mg^2+^-ATP. Thus, Glu-211 of FliI_CAT_ acts as the catalytic glutamate.

Arg-373 in the α subunit of the F_1_ ATPase, which corresponds to Arg-374 of FliI, functions as the arginine finger that protrudes into the nucleotide-binding site of the adjacent β-subunit. The side chain of this arginine residue forms a positively charged binding pocket for the negative charge of the γ-phosphate of ATP (Rees et al., 2012). The *fliI(R374A)* mutation inhibits FliI ring formation significantly and decreases ATPase activity. This effect indicates that Arg-374 of FliI stabilizes the binding of ATP to the P-loop in a way similar to Arg-373 of the α subunit. These observations suggest that FliI and the F_1_ ATPase share a similar catalytic pathway for ATP hydrolysis.

The binding of ADP to the P-loop induces a conformational change in FliI_C_ relative to FliI_CAT_, suggesting that the FliI hexamer may undergo conformational changes in its C-terminal domains that are coupled with the catalytic reaction cycle in the same way as in the F_1_ ATPase. This idea is supported by the asymmetric cryoEM structure of the SctN_6_-SctO_1_ ring complex with a non-hydrolyzable ATP analog (Majewski et al., 2019).

FliJ and its vT3SS homolog SctO adopt an antiparallel coiled-coil structure that is similar to the two-stranded α-helical coiled-coil part of the γ subunit of the F_1_ ATPase (Figure 5D; Ibuki et al., 2011). FliJ binds to the C-terminal region of the first α-helix of FliI_C_ (residues 382–406 of *Salmonella* FliI), which corresponds to the region of the β subunit that is responsible for interaction with the γ subunit. This interaction facilitates FliI ring formation and increases the ATPase activity of FliI. FliJ penetrates the central cavity of the FliI_6_ ring like the γ subunit in the F_1_ ATPase (Figure 5C). These observations have been confirmed by the cryoEM structure of the SctN_6_-SctO_1_ ring complex. FliJ has been shown to exert a rotor-like function in both rotary F_1_ and V_1_ ATPases (Kishikawa et al., 2013; Baba et al., 2016). Thus, the FliI_6_-FliJ_1_ ring complex may function as an ATP-driven rotary motor that couples ATP hydrolysis with the rotation of FliJ within the FliI hexamer.

### Peripheral Stalk of Flagellar ATPase Ring Complex

**FIGURE 6:**
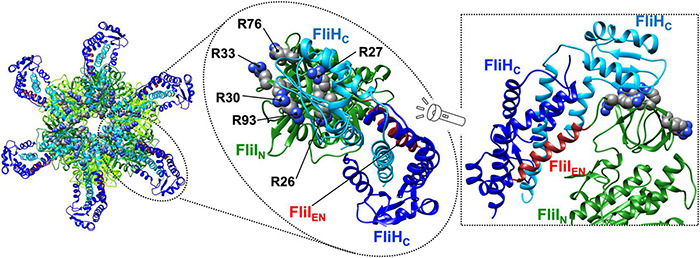
Atomic model of the FliH_C12_-FliI_6_ ring complex. Cα ribbon representation of the FliH_C2_-FliI_1_ complex (PDB ID: 5B0O) is shown. The C-terminal domain of FliH (residues 141–235, FliH_C_) forms a dimer via an interaction of residues 101–140, which adopt a coiled-coil structure. The FliH_C_ dimer binds to each N-terminal domain (FliI_N_) of the FliI_6_ ring. Interestingly, one FliH_C_ domain (blue) binds to the N-terminal α-helix consisting of residues 2–21 of FliI (brown, FliI_EN_), and the other (cyan) binds to a positively charged region formed by R26, R27, R30, R33, R76, and R93 of FliI. These two domains adopt conformations that are completely different from each other.

The b and δ subunits of the F_O_F_1_ ATP synthase form the peripheral stalk that connects the α_3_β_3_γ_1_ε_1_ ring complex to the membrane-embedded F_O_ unit (Figure 1). The extreme N-terminal region of the b subunit binds to F_O_, whereas the δ subunit interacts with the extreme N-terminal region of the α subunit of F_1_ (Walker and Dickson, 2006). The N-terminal and C-terminal regions of FliH and its vT3SS homolog SctL are homologous to the b and δ subunits of the ATP synthase (Pallen et al., 2006). This is confirmed by the crystal structure of an N-terminally truncated variant of *Salmonella* FliH consisting of residues 99–235 in complex with FliI (Figure 6; Imada et al., 2016).

*Salmonella* FliH consists of 235 amino-acid residues and forms a homo-dimer through residues 101–140, which form a coiled-coil structure (Minamino and Macnab, 2000b; González-Pedrajo et al., 2002). The FliH dimer binds to each FliI_N_ domain of the FliI_6_ ring (Figure 6, left panel) and also to the FliN protein in the C-ring (González-Pedrajo et al., 2006; McMurry et al., 2006; Paul et al., 2006). The interactions of FliH with FliN and FliI_N_ are required for efficient and robust association of the FliI_6_-FliJ ring complex with the flagellar basal body (Figure 1; Minamino et al., 2009). The N-terminal domain of FliH (residues 1–140, FliH_N_) adopts a quite elongated α-helical coiled coil structure similar to that of the b subunit of the ATP synthase, and the extreme N-terminal region of FliH is involved in the interaction with FliN (Hara et al., 2012). Both C-terminal domains (residues 141–235, FliH_C_) in the FliH dimer are involved in the interaction with FliI (Minamino et al., 2002). These two FliH_C_ domains have completely different conformations; one binds to the extreme N-terminal α-helix of FliI consisting of residues 2–21, and the other binds to a positively charged cluster consisting of Arg-26, Arg-27, Arg-30, Arg-33, Arg-76, and Arg-93 of FliI_N_ (Figure 6, middle and right panels). Because FliI cannot localize to the flagellar base in the absence of FliH, FliH seems to act as a peripheral stalk to firmly anchor the FliI_6_-FliJ_1_ ring complex to the C-ring.

### Mechanism of Gate Activation

The PMF consists of the electric potential difference (Δψ) and the proton concentration difference (ΔpH) across the cytoplasmic membrane. When the cytoplasmic ATPase ring complex works properly for flagellar assembly, the transmembrane export gate complex uses the Δψ component to drive H^+^-coupled protein export under a variety of environmental conditions (Paul et al., 2008; Minamino et al., 2011, 2021b). However, when the ATPase ring complex becomes non-functional under certain physiological conditions, the export gate complex prefers to use the SMF over a wide range of external pH, indicating that the transmembrane export complex is intrinsically a dual-fuel export engine that can use either H^+^ or Na^+^ as the coupling ion (Minamino et al., 2016b,2021a). This in turn suggests that the cytoplasmic ATPase ring complex switches the export gate complex from the dual-fuel engine mode to a highly efficient Δψ-driven one.

FliJ binds to FlhA_L_ with high affinity to activate the H^+^ channel of FlhA_TM_ and to unlock the entrance gate of the polypeptide channel. As a result, the export gate complex becomes an active H^+^/protein antiporter that couples inward-directed H^+^ flow through the FlhA ion channel with outward-directed protein translocation across the polypeptide channel (Minamino et al., 2011). An inactive export gate complex can also be activated by an increase in Δψ above a certain threshold through an interaction between FliJ and FlhA_L_, suggesting that Δψ is required for efficient and stable interaction between FliJ and FlhA_L_ (Minamino et al., 2021b).

A helix-loop-helix formed by Gln-38, Leu-42, Tyr-45, Tyr-49, Phe-72, Leu-76, Ala-79, and His-83 of FliJ, which are highly conserved residues in FliJ homologs, extends out of the FliI_6_-ring (Figure 5D; Ibuki et al., 2013). This is confirmed by the cryoEM structure of the SctN_6_-SctO_1_ ring. Tyr-45, Tyr-49, and Phe-72 of FliJ are also conserved between FliJ and the γ subunit of the F_1_ ATPase. Among these conserved, surface-exposed residues of FliJ, Phe-72 and Leu-76 are critical for the interaction with FlhA_L_. Residues in the γ subunit corresponding to Phe-72 and Leu-76 are involved in the interaction with an α-helix of the δ subunit of the bovine mitochondrial F_1_ ATPase, which is a homolog of the conserved ε subunit in the F_1_ ATPase family (PDB ID: 1E79) (Gibbons et al., 2000). The *flhA(E351A/W354A/D356A)* triple mutation significantly reduces the binding affinity of FlhA_L_ for FliJ (Inoue et al., 2021). Because the residues from Val-349 to Val-357 of FlhA form an α-helix, FliJ may bind to this α-helix in FlhA_L_ as is seen in the γ-δ interaction in the bovine mitochondrial F_1_ ATPase.

### Mechanistic Role of ATP Hydrolysis for Flagellar Protein Export

ATP hydrolysis by the FliI ATPase and rapid protein translocation by the export complex are both linked to efficient H^+^ translocation through the FlhA ion channel (Morimoto et al., 2016). Recently, it has been reported that ATP hydrolysis by the FliI ATPase also unlocks the entrance gate of the polypeptide channel formed by FliP, FliQ, and FliR for efficient entry of export substrates into the channel (Kinoshita et al., 2021). Furthermore, the *Salmonella* Δ*fliHIJ flhB(P28T) flhA(T490M)* mutant has been isolated as a revertant of the Δ*fliHIJ* mutant that has increased motility (Minamino et al., 2021a). The protein-export activity of the transmembrane export complex in cells with both the *flhA(T490M)* and *flhB(P28T)* mutations is almost at the wild-type level under a variety of experimental conditions even in the absence of the FliH_12_-FliI_6_-FliJ_1_ ring complex (Minamino et al., 2021b). This finding suggests that the export complex normally requires the FliH_12_-FliI_6_-FliJ_1_ complex to serve as a H^+^-coupled protein transporter. Because FliJ requires FliH and FliI to bind efficiently to FlhA_L_ (Minamino et al., 2011), this observation raises the question of how this ATPase ring complex activates the export complex.

The conserved Glu-211 residue of FliI catalyzes ATP hydrolysis. The E211D substitution decreases FliI ATPase activity by about 100-fold (Minamino et al., 2014). *Salmonella* wild-type cells produce an average of 4.4 ± 1.6 flagellar filaments per cell. In contrast, more than 90% of *Salmonella fliI(E211D)* cells have an average of 2.3 ± 1.5 flagellar filaments, and the average length of those filament is only half that of the wild type. Because the fT3SS transports 20,000–30,000 flagellin molecules per flagellum to form a 10–15 μm long helical filament (Minamino and Namba, 2004), the rate of ATP hydrolysis by the FliI ATPase cannot determine the rate of filament assembly, as ATP consumption by the fT3SS during flagellar assembly must be relatively small. Because the export complex still transports flagellar axial proteins even with the infrequent ATP hydrolysis provided by FliI(E211D), ATP hydrolysis appears to be required only for activation of the H^+^-driven export engine.

**FIGURE 7:**
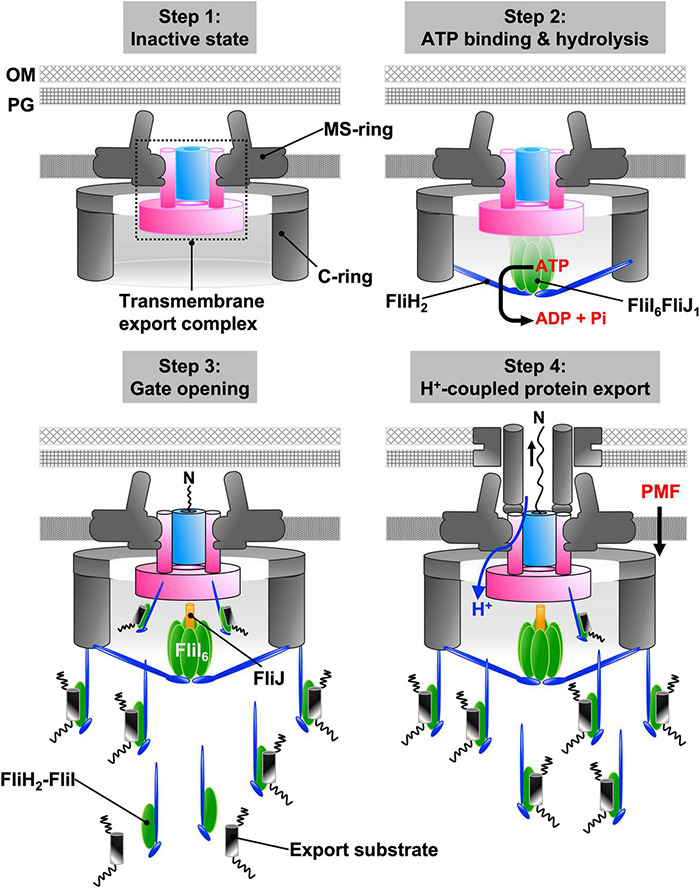
Energy coupling mechanism of the fT3SS. The transmembrane export complex remains inactive until the cytoplasmic ATPase ring complex is formed at the base of the flagellum; the polypeptide and proton channels of the export complex remain closed (step 1). When ATP hydrolysis by FliI induces the rotation of FliJ in the FliI_6_ ring, interactions between FliJ and FlhA_L_ induces conformational rearrangements of the export complex. As a result, the complex becomes an active protein transporter (step 2). The cytoplasmic FliH_2_-FliI_1_ complex acts as a dynamic carrier to deliver export substrates from the cytoplasm to the export complex (step 3). Upon docking of an export substrate to the entrance gate of the polypeptide channel, the gates of both polypeptide and proton channels are opened. The export complex can now act as a H^+^/protein antiporter that couples H^+^ flow through the ion channel with protein translocation into the polypeptide channel (step 4).

The six FliI_C_ domains undergo cooperative and sequential conformational changes triggered by ATP hydrolysis. Deletion of residues 401–410 in the first α-helix of FliI_C_, which is responsible for the interaction with FliJ, significantly decreases the protein transport activity of the fT3SS although the ATPase activity is still at about 40% of the wild-type level. Because this deletion does not inhibit the interaction between FliI and FliJ, it may affect conformational changes in the FliI_C_ domains that rotate FliJ within the FliI hexamer. Rotation of FliJ may induce conformational changes in the FlhA_TM_ domain through an interaction between FliJ and FlhA_L_, thereby activating the FlhA ion channel and unlocking the entrance gate of the polypeptide channel (Figure 7).

The elementary step size of γ rotation within the α_3_β_3_ ring is 120°, which is composed of 80° and 40° sub-steps driven by ATP binding–ADP release and ATP hydrolysis–Pi release, respectively (Watanabe and Noji, 2013). The cryoEM structure of the SctN_6_-SctO_1_ ring complex has suggested a possible rotational mechanism for catalysis. In this model, the SctO stalk rotates in the SctN_6_ ring through an interaction between each SctN_C_ domain and SctO. Because the SctN_6_-SctO_1_ ring complex has six catalytic sites, the elementary step size of SctO rotation within the SctN_6_ ring is probably 60° (Majewski et al., 2019). The FliI(E211Q) substitution in *Salmonella*, which completely eliminates ATPase activity but not ATP binding to the P-loop of FliI_CAT_, results in only 17% of cells having one or two flagellar filaments about 25% the length of those of the wild type (Minamino et al., 2014). Thus, ATP binding to the P-loop is sufficient to activate the H^+^-driven export engine of the fT3SS to some degree. So, the 60° rotation of FliJ may be divided into two sub-steps, and ATP binding may induce the first sub-step, which may be sufficient to activate the H^+^-driven export engine weakly.

### The Heterotrimeric FliH_2_-FliI_1_ Complex Acts as Dynamic Carrier

The FliI monomer interacts with the FliH dimer to form a hetero-trimer in the cytoplasm (Minamino and Macnab, 2000b; Auvray et al., 2002). High-resolution imaging of fluorescently labeled FliI *in vivo* has revealed that FliH_2_-FliI_1_ complexes are associated with the basal body through interactions of FliH with FlhA and FliN. FliI-YFP shows a rapid exchange between the basal body and a freely diffusing cytoplasmic pool. The FliI(K188I) substitution, which inhibits ATP binding to the P-loop in FliI_CAT_, does not affect the exchange rate of FliI-YFP, suggesting that ATP hydrolysis does not drive the association-dissociation cycle (Bai et al., 2014). FliH also suppresses the ATPase activity of the FliH_2_-FliI_1_ complex (Minamino and Macnab, 2000b). Deletion of *flhA* decreases the number of FliI-YFP molecules associated with the basal body but does not affect the exchange rate. The highly conserved Trp-7 and Trp-10 residues of FliH_N_ are directly involved in the interactions of FliH with FliN and FlhA_TM_ (Hara et al., 2012; Notti et al., 2015). Because the interaction between FliH and FliN is required for efficient localization of the FliH_2_-FliI_1_ complex to the flagellar base, the FliH-FliN interaction must be highly dynamic to achieve rapid and efficient flagellar protein export by the fT3SS. Flagellar chaperones in complex with their cognate substrates both bind to FliI_C_, suggesting that FliI_C_ is also involved in substrate recognition (Thomas et al., 2004; Imada et al., 2010; Minamino et al., 2012b). Pull-down assays have demonstrated that chaperone-associated export substrates bind to FlhA_C_ and FlhB_C_ even in the absence of FliHI (Evans et al., 2013; Kinoshita et al., 2013; Inoue et al., 2019). However, they require the FliH_2_-FliI_1_ complex to efficiently interact with FlhA_C_ and FlhB_C_ *in vivo* (Minamino et al., 2016b; Inoue et al., 2018; Kinoshita et al., 2021).

*In vitro* protein transport assays using inverted membrane vesicles have shown that addition of the purified FliH_2_-FliI_1_ complex considerably increases the transport of flagellar axial protein into the lumen of the membrane vesicles (Terashima et al., 2018, 2020). Thus, the FliH_2_-FliI_1_ complex acts as a dynamic carrier to deliver chaperone-associated export substrates from the cytoplasm to the flagellar base and to facilitate their docking to FlhA_C_ and FlhB_C_, thereby allowing the activated export complex to unfold and transport export substrates into the central channel of the flagellum.

### The FliH_2_FliI Complex Is Required for Efficient Flagellar Assembly

*Salmonella* cells lacking the FliH and FliI proteins display a very weak motile phenotype. This defect is considerably alleviated by either an increase in the expression level of export substrates and chaperones or an increase in the PMF (Erhardt et al., 2014). Expression of *Vibrio alginolyticus* FlhA, which has 73.2% similarity and 52.9% identity in amino acid sequence with *Salmonella* FlhA, restores motility in the *Salmonella* Δ*flhA* mutant but does not increase motility in the *Salmonella* Δ*fliHI flhB(P28T)*Δ*flhA* mutant (Minamino et al., 2016a). Thus, *Vibrio* FlhA requires FliH and FliI to perform protein export in the *Salmonella* fT3SS. Deletion of *flgM*, which encodes the negative regulator of the flagellar regulon, increases the expression levels of FliJ, export substrates and flagellar export chaperones and allows *Vibrio* FlhA to perform protein transport even in the absence of FliH and FliI. These results suggest that FlhA needs FliH and FliI to buffer protein export against internal perturbations (Minamino et al., 2016a).

The fT3SS utilizes the secreted molecular ruler protein FliK to stop growth of the hook at about 55 nm (Minamino et al., 1999; Erhardt et al., 2011; Kinoshita et al., 2017). The Δ*fliHI flhB(P28T)* bypass mutant cannot properly control the length of the hook, although it secrets the hook capping protein FlgD and the FliK ruler into the culture media almost at the wild-type level (Inoue et al., 2018). However, secretion level of the hook protein FlgE is about 10-fold lower than the wild-type level. The *flhA(F459A)* mutation, which targets a residue within FlhA_C_ (Figure 4), significantly increases the secretion of FlgE, so the secreted FliK ruler can measure the length of the hook more precisely. Neither the secretion levels nor control of hook length is affected by the FlhB(P28T) and FlhA(F459A) substitutions when FliH and FliI are present. Because FlgD, FlgE, and FliK bind to FliH and FliI as well as FlhA_C_ and FlhB_C_ (Minamino and Macnab, 2000c), the FliH_2_-FliI complex may coordinate targeting of FlgD, FlgE, and FliK to the FlhA_C_-FlhB_C_ docking platform to make control of the hook length more robust.

FlgN, FliS, and FliT act as export chaperones for FlgK/FlgL, FliC, and FliD, respectively (Fraser et al., 1999; Auvray et al., 2001). The chaperone-substrate complexes bind FlhA_C_ with nanomolar affinity (Figure 4; Kinoshita et al., 2013). This strong interaction of the chaperone with FlhA_C_ facilitates protein unfolding and transport by the H^+^-driven export complex (Furukawa et al., 2016; Minamino et al., 2021c). In wild-type cells, more than 90% of flagellin molecules transported by the fT3SS assemble into the filament. The Δ*fliHI flhB(P28T) flhA(F459A)* cannot efficiently produce the hook-filament junction and filament cap structures at the hook tip, and hence more than 90% of the flagellin molecules are secreted as monomer into the culture supernatant. Because FlgN and FliT bind to the FliH_2_-FliI_1_ complex whereas FliS does not (Thomas et al., 2004; Minamino et al., 2012b; Sajó et al., 2014), the FliH_2_-FliI_1_ complex may contribute to hierarchical targeting of the flagellar chaperones to FlhA_C_, thereby allowing the junction and filament cap structures to be efficiently formed at the hook tip prior to filament formation. Thus, the FliH_2_-FliI_1_ complex works with the FlhA_C_-FlhB_C_ docking platform to ensure the correct order of protein export.

### Energy Coupling Mechanism

The information thus far summarized allows us to propose a model for the energetics of protein export by the fT3SS (Figure 7). The transmembrane export complex remains inactive until the cytoplasmic ATPase ring complex localizes to the flagellar base through an interaction between FliH_N_ and FlN (Step 1). ATP hydrolysis by the FliI ATPase induces the rotation of FliJ within the FliI_6_-ring at the FlhA_C_-FlhB_C_ docking platform. The interactions of FliJ and FlhA_L_ induce conformational changes in the export complex that activate the FlhA ion channel and unlock the entrance gate of the polypeptide channel (Step 2). Then, cytoplasmic FliH_2_-FliI_1_ complexes escort export substrates and chaperone-substrate complexes from the cytoplasm to the FlhA_C_-FlhB_C_ docking platform through the interactions of FliH with FliN and FlhA (Step 3). The binding of the export substrate to the docking platform induces opening of the gate to the polypeptide channel, and the activated export complex acts as an H^+^/protein antiporter that couples proton flow through the FlhA ion channel with the translocation of export substrates into the polypeptide channel (Step 4). The association-dissociation cycle of the FliH_2_-FliI complex with the docking platform allows the transport of flagellar axial proteins in a highly controlled manner.

## Conclusion and Perspectives

The transmembrane export complex of the fT3SS is a dual-fuel export engine that uses either H^+^ or Na^+^ as the coupling ion to drive export of flagellar proteins. Interestingly, when the cytoplasmic ATPase ring complex works properly, the export gate preferentially utilizes the PMF to drive H^+^-coupled protein export. FlhA_TM_ acts as a dual ion channel to conduct both H^+^ and Na^+^. Because there is not yet structural information about the FlhA_TM_, it remains unknown how the cytoplasmic ATPase ring complex switches the ion channel mode of FlhA_TM_ from an inefficient dual-ion channel to a highly efficient H^+^ channel.

The export complex couples inward-directed ion flow through FlhA with outward-directed protein translocation through the polypeptide channel. Recently, the cryoEM structure of the FliP_5_-FliQ_4_-FliR_1_ complex associated with the basal body has been obtained with near atomic level (Johnson et al., 2021; Tan et al., 2021). Unfortunately, both FlhA and FlhB are lacking in the structure. To clarify the energy coupling mechanism, high-resolution structures of the entire export complex in different states of substrate export will be required.

The entire structure of the FliI_6_-FliJ_1_ ring complex looks similar those of rotary F_1_ and V_1_ ATPases. The FliI_6_-FliJ_1_ ring complex hydrolyzes ATP at the interfaces between FliI subunits and may induce sequential and cooperative conformational changes in FliI_C_, which is involved in the interaction with FliJ. These observations lead to the hypothesis that ATP hydrolysis by the FliI ATPase presumably allows FliJ to rotate within the FliI_6_ ring. This idea is supported by the cryoEM structure of the SctN_6_-SctO_1_ ring complex. To demonstrate the rotational catalytic mechanism of the FliI_6_-FliJ_1_ ring complex directly will require a biophysical approach.

The fT3SS transports fourteen different flagellar proteins in their copy numbers ranging from a few to tens of thousands in a sequential manner so that the flagellum can be built efficiently. The fT3SS must ensure the correct order of export of flagellar proteins for this to be an efficient process. The FliH_2_-FliI_1_ complex is required for efficient and robust flagellar assembly. Although an *in vitro* protein transport assay using inverted membrane vesicles has been established for the fT3SS, a quantitative measurement of ordered flagellar protein export will be needed to understand how the FliH_2_-FliI_1_ complex contributes to hierarchical protein targeting to the export complex.

## Author Contributions

TM, MK, and KN researched and wrote the review article. All authors contributed to the article and approved the submitted version.

## Conflict of Interest

The authors declare that the research was conducted in the absence of any commercial or financial relationships that could be construed as a potential conflict of interest.

## Publisher’s Note

All claims expressed in this article are solely those of the authors and do not necessarily represent those of their affiliated organizations, or those of the publisher, the editors and the reviewers. Any product that may be evaluated in this article, or claim that may be made by its manufacturer, is not guaranteed or endorsed by the publisher.
